# Fetal autonomic responses inferred from heart rate variability during recurrent decelerations without acidosis—An observational study

**DOI:** 10.1002/ijgo.71066

**Published:** 2026-05-11

**Authors:** Shoichi Magawa, Masafumi Nii, Yuya Tamaishi, Asumi Okumura, Sho Takakura, Shintaro Maki, Kuniaki Toriyabe, Erina Takayama, Eiji Kondo, Tomoaki Ikeda

**Affiliations:** ^1^ Department of Obstetrics and Gynecology Mie University Graduate School of Medicine Tsu Japan

**Keywords:** autonomic nervous system, fetal heart rate, heart rate variability, labor, obstetric, parasympathetic nervous system

## Abstract

**Objective:**

Recurrent fetal heart rate (FHR) decelerations without acidosis are frequently observed during labor; however, the underlying autonomic mechanisms remain incompletely understood. This study explored fetal autonomic responses to recurrent decelerations using FHR and FHR variability (FHRV) parameters.

**Methods:**

This retrospective observational study included healthy women with singleton pregnancies, no pregnancy complications requiring treatment, and an umbilical artery pH >7.2 at delivery. Continuous FHR monitoring using fetal head electrodes was performed for ≥2 h before delivery (June–December 2021). FHRV was assessed using time‐domain, frequency‐domain, and nonlinear indices.

**Results:**

Twenty‐four patients were included. Baseline FHR did not differ between groups. In the recurrent deceleration group, root mean square of successive differences and high‐frequency power were higher during the final 60 min before delivery. No differences were observed in standard deviation of normal‐to‐normal intervals or very‐low‐frequency power. Sample entropy was also higher, suggesting increased complexity of autonomic regulation.

**Conclusion:**

Fetuses with recurrent decelerations without acidosis demonstrated patterns consistent with increased vagal modulation, as inferred from FHRV indices. These findings may reflect an adaptive autonomic response; however, given the exploratory nature and small sample size, further studies are warranted.

## 1 INTRODUCTION

Recurrent fetal heart rate (FHR) decelerations without associated acidosis are among the most commonly observed features during intrapartum FHR monitoring. In the first stage of labor, uterine contractions occur at a relatively low frequency of approximately twice per 10 min (once every 5 min). After the first stage of labor, the frequency increases to four or five times per 10 min (once every 2.5 min).^1^ These transient changes, representing the mildest form of stress experienced by the fetus during labor, raise important physiologic questions. How does the fetus respond to such stress? Although sympathetic activation is the predominant response to stressors in adults,^2^ whether the fetal autonomic nervous system exhibits a similar pattern or a distinct regulatory mechanism remains unclear.

During labor, uterine contractions intermittently reduce uteroplacental perfusion, cause umbilical cord compression, and compress the fetal head against the birth canal, leading to brief episodes of mild hypoxia. Here, we explored how the fetal autonomic nervous system responds to recurrent decelerations by examining FHR and FHR variability (FHRV) parameters in normal pregnancies. Using time‐domain, frequency‐domain, and nonlinear analyses, we sought to clarify whether the fetal response to mild hypoxia was characterized by sympathetic activation, as observed in adults, or by a pattern consistent with vagal predominance. Understanding the functional significance of these autonomic changes may provide insights into fetal resilience, such as the anti‐inflammatory response,^3^, ^4^, ^5^ which has recently gained attention and improved the interpretation of FHR monitoring during labor. Therefore, this exploratory observational analysis characterized FHRV patterns during recurrent decelerations without acidosis. Rather than testing a predefined mechanistic hypothesis, we aimed to identify physiologic patterns that may inform future hypothesis‐driven research and improve interpretation of intrapartum monitoring.

## 2 MATERIALS AND METHODS

### 2.1 Study design

This was a single‐center retrospective observational study conducted at a regional tertiary referral center. Given its exploratory nature, the analyses were primarily descriptive and intended to identify potential physiologic patterns rather than establish causal mechanisms.

### 2.2 Study population

This study was exploratory in nature, so the sample size was determined by the number of eligible cases meeting strict inclusion criteria during the study period.

We examined deliveries at 37 weeks or more of pregnancy at Mie University Hospital between June and December 2021. All deliveries within the study period were screened, followed by predefined exclusions. Eligible cases were categorized into recurrent and non‐recurrent deceleration groups, followed by FHRV analysis.

We included singleton pregnancies, without complications requiring treatment during pregnancy, and with an umbilical artery pH greater than 7.2 at delivery. We excluded cases of fetal growth restriction (an estimated fetal weight below −1.5 standard deviations on transabdominal ultrasound examinations performed during pregnancy), congenital anomalies causing fetal hypoxia or growth restriction (especially fetal cardiac disease), and placenta previa (placental location within 20 mm of the internal cervical os after 30 weeks of pregnancy). Continuous FHR monitoring using fetal head electrodes was required for at least 2 h before delivery to ensure uninterrupted data collection (Figure 1).

**FIGURE 1:**
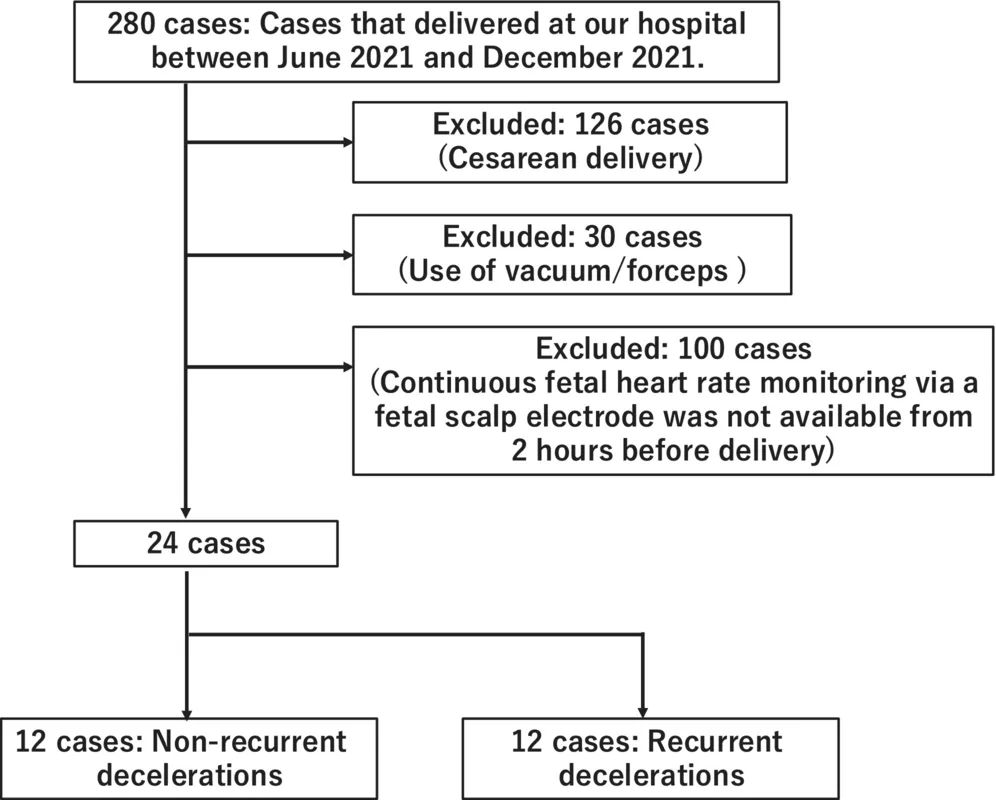
Flow diagram of study groups. The figure shows the patients observed at our hospital between June and December 2021 in whom fetal heart rate monitoring was continuously performed using fetal head electrodes 2 h before delivery.

FHR and clinical data were retrospectively extracted after delivery from the electronic medical records and monitoring system.

### 2.3 Definition of recurrent decelerations

Recurrent decelerations were defined as at least three uterine contractions within 10 min, accompanied by late and/or variable decelerations in more than 50% of these contractions, lasting longer than 20 min, and directly leading to delivery.

Participants were classified according to the above criteria as those who developed recurrent decelerations before delivery (Recurrent) and those who did not (Non‐recurrent). Group allocation was determined by one of the authors (SM) based on FHR monitoring tracings at delivery. All FHR patterns were evaluated based on the National Institute of Child Health and Human Development guidelines.^6^

### 2.4 Clinical management

After 37 weeks of pregnancy, patients were admitted when they experienced painful uterine contractions less than 10 min apart. Upon admission, continuous transabdominal FHR monitoring was performed. The FHR was recorded using external monitoring or a scalp electrode, and uterine activity was assessed using tocodynamometry. Following spontaneous or artificial rupture of the membranes, a fetal head electrode and an intrauterine pressure sensor were placed in the uterine cavity. The fetal electrocardiogram and RR intervals were recorded and stored. The obstetrician promptly collected umbilical cord blood from the umbilical artery after delivery, and blood gas analysis was performed using a RAPID Point 500 (Siemens Healthcare, Camberley, UK).

### 2.5 FHRV analysis

Fetal electrocardiogram signals were obtained using fetal spiral electrodes, and continuous RR intervals were extracted using MemCalc/BonalyLight (GMS Co., Ltd., Tokyo, Japan). An experienced obstetrician (SM) manually removed FHR decelerations from the RR interval trace. The FHRV analysis excluded regions affected by intrapartum deceleration. After this, for FHRV data, values above 60 min were excluded. FHRV parameters were assessed similarly, using time‐series data reconstructed after removing deceleration regions from continuous monitoring, starting 2 h before delivery. Data from 60 min before delivery were compared with those collected every 5 min from that point to delivery. The RR intervals were imported into Kubios Standard HRV (v3.0.2, Kuopio, Finland), where deceleration areas were removed before analysis. All indices were assessed over 1‐min continuous epochs and averaged at 5‐min intervals.

Time‐domain measures included the standard deviation of RR intervals (SDNN) and the root mean square of successive RR intervals (RMSSD). The SDNN was calculated as the SD of all RR intervals within each epoch, providing a measure of the total FHRV. The RMSSD was calculated as the RMS of successive RR intervals during each epoch by measuring the beat‐to‐beat FHRV, which is particularly sensitive to high‐frequency oscillations.^7^, ^8^

Frequency‐domain analysis was conducted using a Fast Fourier Transform spectrum with Welch periodogram, employing an interpolation rate of 4 Hz and a 50% window overlap. The frequency bands analyzed included very low frequency (VLF; 0–0.04 Hz), low frequency (LF; 0.04–0.15 Hz), and high frequency (HF; 0.15–0.4 Hz).^7^, ^9^, ^10^

For nonlinear analysis, sample entropy (SampEn) was measured to assess the complexity of FHR patterns using a vector size of m = 2 and a tolerance of r = 0.2.^11^, ^12^, ^13^

### 2.6 Statistical methods

Paired *t*‐tests were performed using SPSS version 27 (IBM Corporation, Armonk, NY, USA). The FHR was assessed by comparing the epochs to the baseline FHR value, defined as 120 min before delivery. These variables were compared between the two groups using the Mann–Whitney *U* test. Statistical significance was set at a *P* value less than 0.05.

### 2.7 Ethical approval

This study was conducted in accordance with the principles of the Declaration of Helsinki. The Institutional Review Board of the Mie University Hospital approved the study protocol (No. H2021‐232, December, 22 021) and waived the need for informed consent because of the retrospective analysis of anonymized data.

## 3 RESULTS

A total of 24 patients were analyzed in this study (Figure 1). No significant differences were observed between the two groups regarding maternal age, gestational age at delivery, birth weight, or umbilical artery pH (Table 1), indicating that the groups were comparable, with no underlying differences affecting fetal outcomes. In the Non‐recurrent group, maternal comorbidities included mild thyroid dysfunction (two cases) and schizophrenia (one case); one fetus showed intestinal dilatation. In the Recurrent group, mild maternal hypertension at delivery was observed in two cases, mild gestational diabetes mellitus in two cases, and mild thyroid dysfunction in one case. One fetus was suspected of having cerebellar hypoplasia. In both groups, a mixture of variable and late decelerations was observed.

**TABLE 1:** Maternal and neonatal findings.^a^

Characteristics	Non‐recurrent group	Recurrent group	*P*‐value
Maternal			
Number	12	12	
Age, years	36.5 (34.3–38.5)	34.5 (28.3–38.8)	0.242
Primipara patient	9	10	
BMI before delivery	24.9 (22.0–28.9)	25.1 (24.0–27.5)	0.932
Neonatal			
Delivery, d	279 (273–282)	279 (276–284)	0.932
Birth weight, g	3134 (2973–3270)	3052 (2744–3506)	0.887
Birth weight, centile	54.3 (37.8–81.0)	67.5 (16.3–90.2)	1.000
Sex (male: female)	5:7	8:4	
Apgar score at 1 min	8 (8–8)	8 (8–8)	1.000
Apgar score at 5 min	9 (9–9)	9 (9–9)	0.378
Umbilical artery blood gas analysis			
pH	7.30 (7.27–7.34)	7.31 (7.27–7.34)	0.630
po_2_, mm Hg	15.6 (14.1–18.9)	17.7 (12.7–21.1)	0.976
pco_2_, mm Hg	40.5 (38.0–44.6)	41.1 (36.8–48.6)	0.713
BE, mEq/L	−6.1 (−8.9 to −4.6)	−5.8 (−7.4 to −3.6)	0.695

Abbreviations: BE, base excess; BMI, body mass index (calculated as weight in kilograms divided by the square of height in meters).

^a^ Values are presented as medians and interquartile ranges unless otherwise stated.

### 3.1 FHR changes

FHR from 2 h before delivery to every 10 min before birth showed no significant differences between the two groups (Figure 2). The mean baseline FHR remained within the normal range in both groups, indicating that recurrent decelerations did not result in an overall increase in baseline heart rate or fetal distress.

**FIGURE 2:**
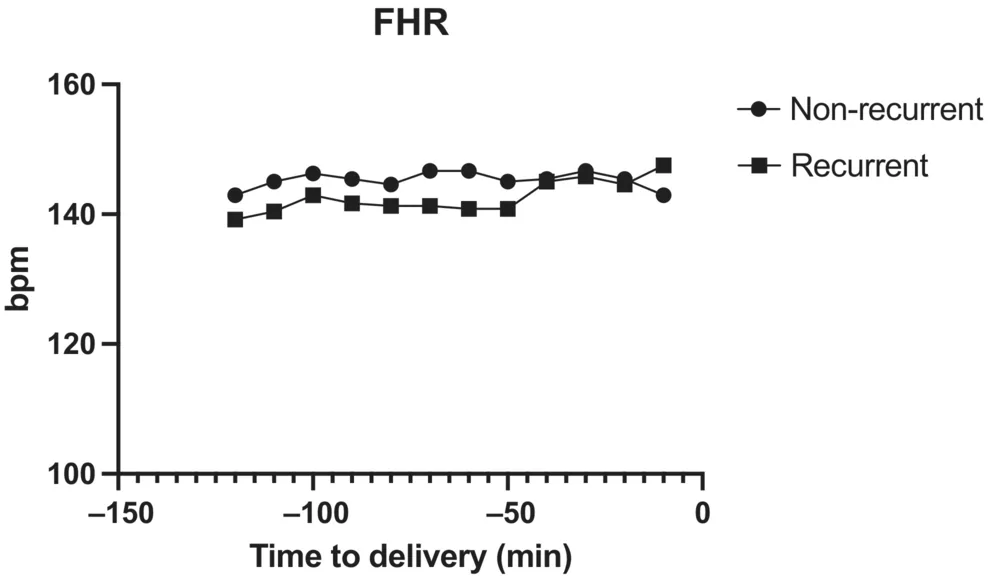
Fetal heart rate (FHR) changes up to delivery—change in FHR baseline from 2 h before delivery in the Recurrent and Non‐recurrent groups. No significant differences existed in the observation periods between the two groups.

### 3.2 FHRV parameter changes

Time‐domain analysis revealed that RMSSD, a measure of short‐term variability (often interpreted as vagal modulation in adults), was significantly higher in the recurrent deceleration group during the last 60 min before delivery (*Z* = 2.02 to *Z* = 3.35, *r* = 0.41 to *r* = 0.68; all *P* < 0.05). No significant differences were found in SDNN, suggesting that overall autonomic activity remained stable between the groups (Figure 3).

**FIGURE 3:**
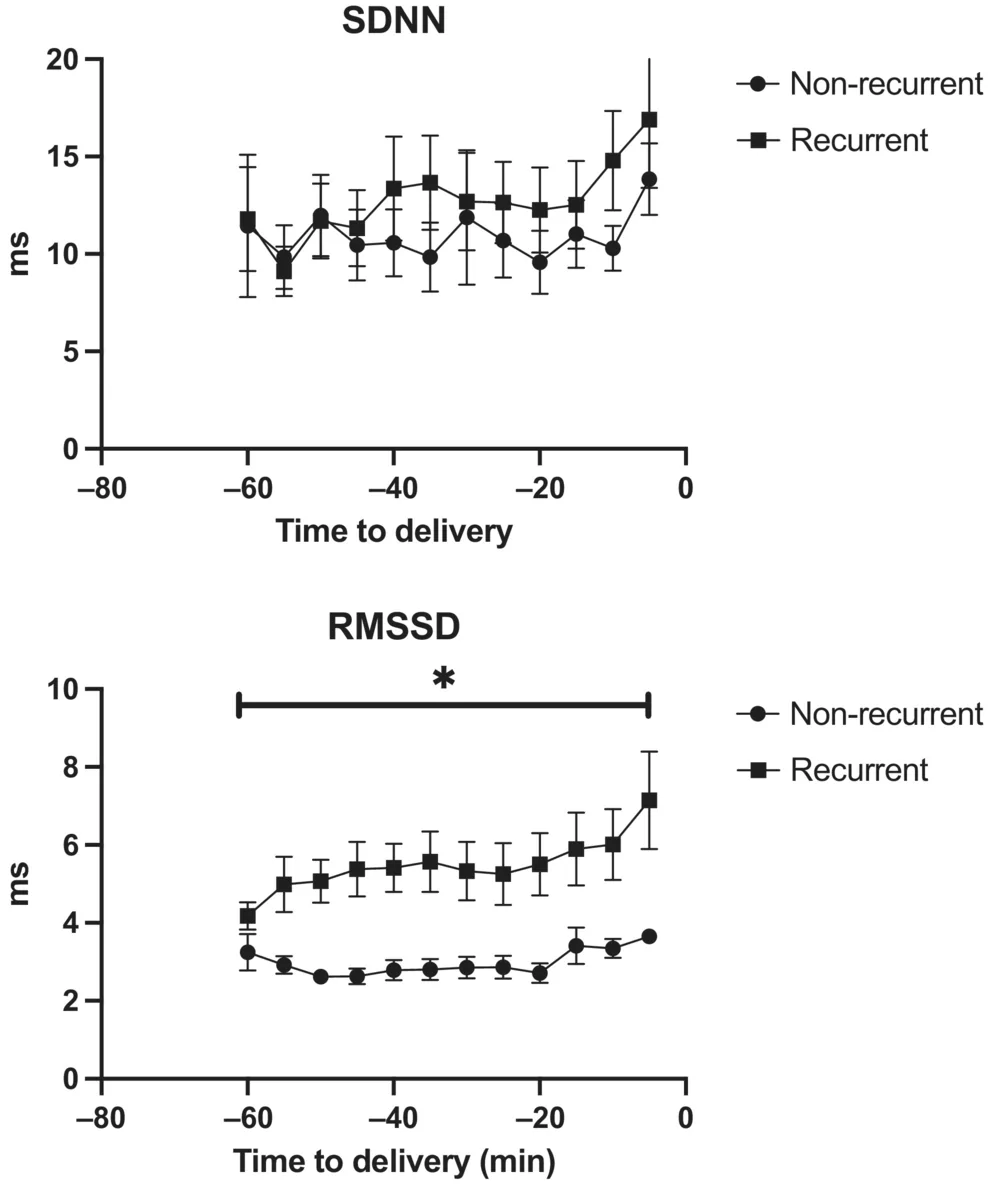
Changes in the time‐domain of fetal heart rate (FHR) variability parameters. The standard deviation of RR intervals and root mean square of successive RR intervals (RMSSD) increased as the delivery approached, with a sustained increase in RMSSD in the Recurrent group compared with that of the Non‐recurrent group.

In the frequency‐domain analysis (Figure 4), the spectral power VLFlog, which is typically linked to long‐term autonomic control and hormonal regulation, showed no significant differences between the groups during the observation period. LFlog, associated with both sympathetic and parasympathetic influences, showed a significant increase in the short term in the recurrent deceleration group at 50–45 min (*Z* = 2.771, *r* = 0.57, *P* < 0.05) and 10–5 min before delivery (*Z* = 2.078, *r* = 0.42, *P* < 0.05). HFlog, which is often interpreted as reflecting vagal modulation (mainly in adults), although not a specific marker, showed significant increases in the recurrent deceleration group at 50–35 min (*Z* = 2.440 to *Z* = 2.708, *r* = 0.50 to *Z* = 0.55; all *P* < 0.05) and 10–5 min before delivery (*Z* = 2.540, *r* = 0.52, *P* < 0.05). In the frequency‐domain analysis, HFlog was partially unmeasurable in five cases within the non‐recurrent group.

**FIGURE 4:**
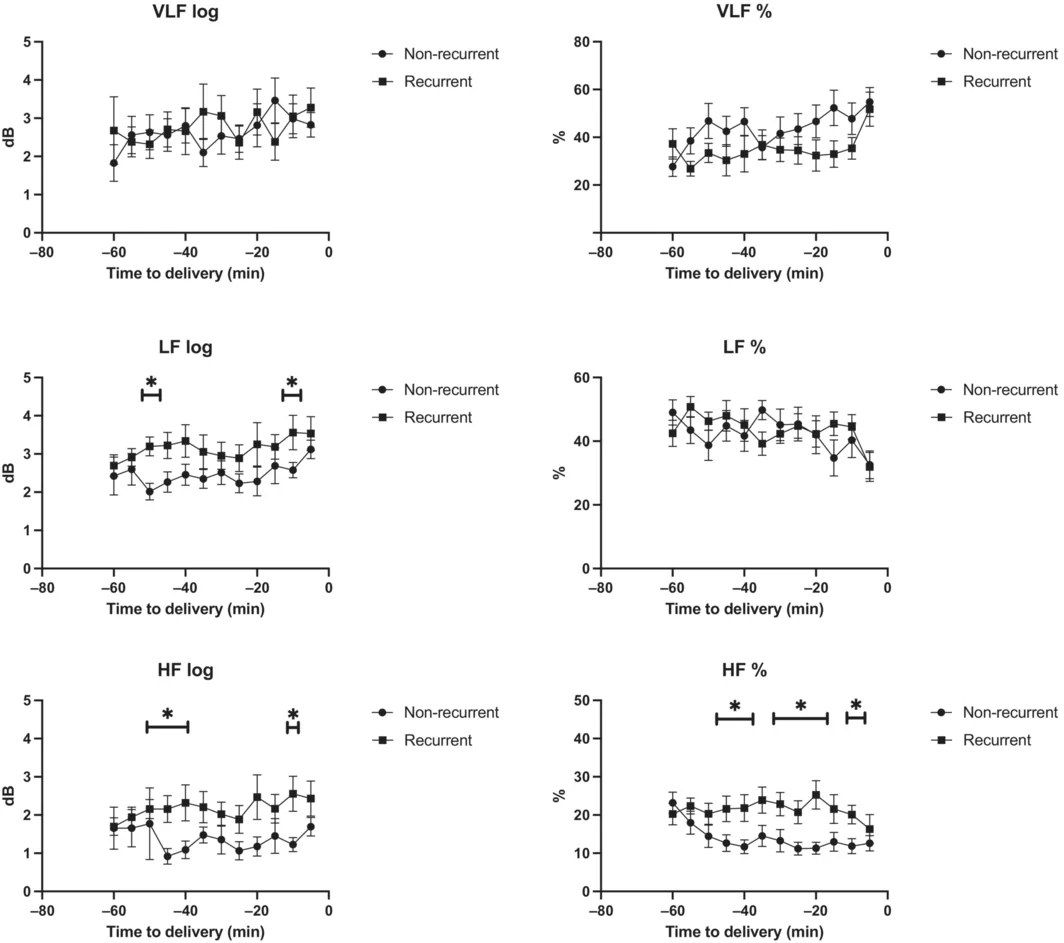
Changes in the frequency‐domain of fetal heart rate variability parameters—a comparison between the Recurrent and Non‐recurrent groups. Particularly, the Recurrent group showed a sustained increase in high‐frequency% in the relative spectrum compared with that of the Non‐recurrent group.

Regarding the relative spectral power (Figure 4), VLF% and LF% showed no significant difference between the groups, whereas HF% showed significant increases in the recurrent deceleration group at 45–35 min (*Z* = 2.078 to *Z* = 2.194, *r* = 0.42 to *r* = 0.45; all *P* < 0.05), 30–15 min (*Z* = 2.021 to *Z* = 2.771, *r* = 0.41 to *r* = 0.57; all *P* < 0.05), and 10–5 min (*Z* = 2.540, *r* = 0.52, *P* < 0.05).

Nonlinear analysis demonstrated that SampEn was significantly higher in the recurrent deceleration group at 55–50 min (*Z* = 2.367, *r* = 0.48, *P* < 0.05) and 15–10 min before delivery (*Z* = 2.367, *r* = 0.48, *P* < 0.05). This suggests greater complexity in FHR regulation and a more adaptive autonomic response to recurrent decelerations (Figure 5).

**FIGURE 5:**
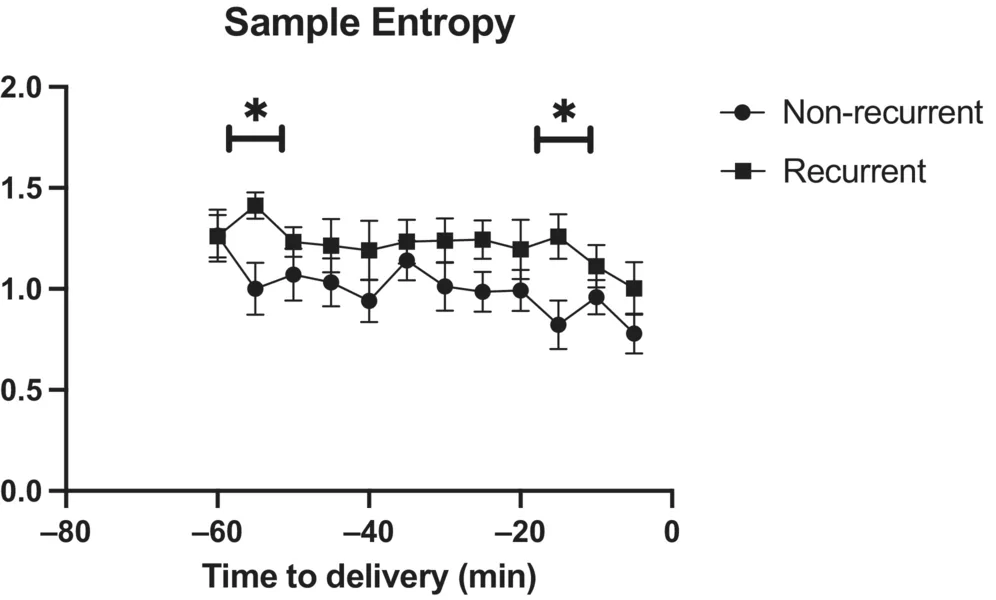
Changes in the nonlinear fetal heart rate variability parameters. The figure shows the changes in Sample Entropy. No significant differences existed in the observation periods between the two groups.

## 4 DISCUSSION

Even in the absence of acidosis during labor, recurrent decelerations associated with uterine contractions are accompanied by changes in fetal autonomic nervous system activity. These changes are reflected in parameters such as RMSSD and HF, which are indicative of vagal modulation in adults (often inferred from HRV indices), suggesting that activation of the fetal parasympathetic nervous system may occur even without acidosis during labor.

This study is the first to evaluate changes in the activity of the fetal autonomic nervous system activity during labor by continuously analyzing fetal electrocardiogram data obtained from human fetuses, demonstrating that data can be appropriately acquired via a fetal scalp electrode and analyzed. These findings suggest the potential to assess the optimal timing for clinical intervention in cases with fetal acidosis or poor neurologic outcomes. However, there are several limitations. The relatively small sample size (n = 24) represents a major limitation, which may affect statistical power and generalizability. The screw‐type fetal scalp electrode cannot be applied in cases with a bleeding risk, and it can only be placed after rupture of membranes. Therefore, in cases of rapidly progressing labor following membrane rupture, adequate risk assessment may be difficult.

This study provides evidence that recurrent decelerations without acidosis are associated with increased parasympathetic activation rather than sympathetic‐driven tachycardia.^14^ Unlike in adults, where stress typically triggers sympathetic activation, the fetal response to transient hypoxia appears to prioritize parasympathetic regulation, likely as an adaptive mechanism to optimize oxygen utilization and maintain cardiovascular stability.^8^, ^15^, ^16^

Considering autonomic modulation in response to recurrent decelerations, the significant increase in RMSSD and HF power in recurrent deceleration cases was consistent with enhanced vagal modulation of the fetal autonomic nervous system, as inferred from HRV indices. This contrasts with the adult response to stress, in which sympathetic activation is typically dominant. The lack of significant changes in SDNN and LF power suggests that the overall autonomic function remains stable, with no indication of fetal distress or compensatory sympathetic overactivity.

Previous human and animal model studies have shown that the parasympathetic nervous system plays a crucial role in regulating FHR during labor. The increase in HF power aligns with findings in fetal sheep, where mild hypoxia is associated with vagal activation and protective cardiovascular adaptations.^17^ The absence of an increase in LF power further suggests that these recurrent decelerations do not trigger a sympathetic response,^18^, ^19^ reinforcing the notion that mild, intermittent hypoxia does not lead to fetal distress but rather to an adaptive autonomic shift.

Alternative physiologic explanations should also be considered. Experimental animal data strongly support a parasympathetic origin.^20^ In fetal sheep exposed to brief repeated umbilical cord occlusions, pharmacologic parasympathetic blockade or vagotomy markedly suppressed FHRV between decelerations, whereas sympathetic pathways did not contribute to inter‐deceleration variability. These findings indicate that vagal activity is the primary regulator of FHRV under labor‐like hypoxic conditions.

The classical interpretation of variable decelerations proposes a baroreflex‐mediated response to cord compression and transient hypertension. Although we cannot completely exclude the possible contribution of the baroreflex in human labor, accumulating evidence suggests a limited role.^21^ Partial cord occlusion does not reliably trigger the baroreflex, and even complete occlusion contributes minimally compared with the peripheral chemoreflex. Instead, fetal hypoxemia activates chemoreceptors and produces vagally mediated decelerations.

Taken together, the increase in RMSSD and HF power observed in this study is most consistent with parasympathetic activation secondary to hypoxia‐induced chemoreflex engagement rather than a baroreflex‐mediated mechanism.

The increase in SampEn observed in the recurrent deceleration group suggests greater complexity and adaptability in fetal autonomic control. Higher SampEn values indicate a more flexible and responsive autonomic nervous system, which may confer resilience to transient hypoxic events during labor. This aligns with previous findings suggesting that increased entropy in FHRV is associated with better neonatal outcomes and a reduced risk of hypoxic–ischemic injury.^22^ However, some reports indicate an increase in entropy due to severe hypoxic–ischemic damage; in this case, a mechanism resulting from severe damage to the autonomic nervous system is assumed to be the underlying cause.^23^

Conversely, conditions such as fetal growth restriction and chronic hypoxia are linked to reduced FHRV complexity, reflecting impaired autonomic regulation.^12^ Our findings suggest that, in healthy pregnancies, recurrent decelerations may be a benign phenomenon, representing a normal autonomic response rather than a sign of fetal compromise.

Understanding the response of the autonomic nervous system to recurrent decelerations is essential for improving clinical decision making during intrapartum monitoring. Traditionally, recurrent decelerations have been considered pathologic findings, often prompting interventions such as forceps delivery or cesarean section. However, such interventions have not consistently led to reduced adverse perinatal outcomes.^24^ Our findings suggest that in the absence of fetal acidosis, these decelerations may reflect physiologic adaptations—such as increased parasympathetic activity and enhanced heart rate variability—rather than fetal distress.

Although this remains speculative, it is conceivable that recurrent decelerations associated with an early shift consistent with increased vagal modulation during labor represent a normal physiologic response. In contrast, as hypoxic–ischemic encephalopathy develops, subsequent decelerations due to myocardial hypoxia may occur, during which the parasympathetic reflex is likely to be blunted. At present, it is difficult to differentiate between parasympathetically mediated recurrent decelerations and those secondary to myocardial hypoxia based solely on macroscopic monitoring patterns. A potential indicator for timely intervention may be the attenuation of previously heightened parasympathetic activity. This hypothesis warrants further investigation. The present findings may provide valuable insight for distinguishing between physiologic and pathologic patterns of recurrent decelerations and so contribute to more appropriate clinical management during labor. Future research should explore how these autonomic responses differ in compromised fetuses, such as those with fetal growth restriction or chronic placental insufficiency. Additionally, investigating how different labor management strategies influence fetal autonomic regulation could provide valuable insights into the optimization of perinatal outcomes.

In conclusion, in fetuses showing recurrent decelerations without acidosis, a pattern consistent with vagal predominance was observed, and its reduction may signal the onset of acidosis, serving as a potential marker for clinical management during labor.

## AUTHOR CONTRIBUTIONS

SM contributed to data curation, formal analysis, methodology, and writing—original draft; MN contributed to conceptualization, data curation, methodology, and writing–original draft; YT contributed to data curation and software; AO contributed to data curation; ST contributed to data curation and supervision; SM and KT contributed to investigation and data curation; ET and EK contributed to investigation and supervision; and TI contributed to project administration, conceptualization, methodology, supervision, writing, review, and editing.

## FUNDING INFORMATION

This work was supported by JSPS KAKENHI (grant number JP 22 K09545).

## CONFLICT OF INTEREST STATEMENT

The authors have no conflicts of interest.

## ETHICS STATEMENT

This study was conducted in accordance with the principles of the Declaration of Helsinki. The Institutional Review Board of the Mie University Hospital approved the study protocol (No. H2021‐232, December 2, 2021) and waived the need for informed consent because of the retrospective analysis of anonymized data.

## DECLARATION OF TRANSPARENCY

The lead author affirms that this manuscript is an honest, accurate, and transparent account of the study being reported; that no important aspects of the study have been omitted; and that any discrepancies from the study as originally planned (if relevant) have been explained.

## Data Availability

The data that support the findings of this study are available on request from the corresponding author. The data are not publicly available due to privacy or ethical restrictions.
